# Targeting immune checkpoints in malignant glioma

**DOI:** 10.18632/oncotarget.12702

**Published:** 2016-10-16

**Authors:** Xuhao Zhang, Shan Zhu, Tete Li, Yong-Jun Liu, Wei Chen, Jingtao Chen

**Affiliations:** ^1^ Institute of Translational Medicine, The First Hospital, Jilin University, Changchun, China; ^2^ Sanofi Research and Development, Cambridge, MA, USA; ^3^ ADC Biomedical Research Institute, Saint Paul, MN, USA

**Keywords:** PD-1/PD-L1, CTLA-4, IDO, malignant glioma, immunotherapy

## Abstract

Malignant glioma is the most common and a highly aggressive cancer in the central nervous system (CNS). Cancer immunotherapy, strategies to boost the bodys anti-cancer immune responses instead of directly targeting tumor cells, recently achieved great success in treating several human solid tumors. Although once considered immune privileged and devoid of normal immunological functions, CNS is now considered a promising target for cancer immunotherapy, featuring the recent progresses in neurobiology and neuroimmunology and a highly immunosuppressive state in malignant glioma. In this review, we focus on immune checkpoint inhibitors, specifically, antagonizing monoclonal antibodies for programmed cell death protein-1 (PD-1), cytotoxic T-lymphocyte-associated antigen-4 (CTLA-4), and indoleamine 2,3-dioxygenase (IDO). We discuss advances in the working mechanisms of these immune checkpoint molecules, their status in malignant glioma, and current preclinical and clinical trials targeting these molecules in malignant glioma.

## INTRODUCTION

Malignant glioma is the most common type (accounting for approximately 80%) of primary malignant brain tumors and associated with exceptionally high morbidity and mortality [1, 2]. The standard therapy for newly diagnosed malignant gliomas involves surgical resection combined with chemotherapy and/or radiotherapy. Although advances in radiotherapy and chemotherapy have brought modest improvements in the survival of patients with malignant glioma, the invasive nature of the disease continue to limit the 5-year survival of glioblastoma (GBM) and its variants to only 4.7% [3–5]. Therefore, there is an urgent need to develop novel therapeutic modalities that specifically target the pathogenesis of malignant gliomas.

Cancer immunotherapy, the idea of boosting the tumor-specific adaptive immune activities instead of directly targeting cancer cells, presents its debut in history more than 100 years ago [6]. After decades of disappointment, it proves its values with recent successes in the treatment of multiple solid and hematological cancers [7]. These successes were built upon incessant efforts to understand the mechanisms underlying cancer immune regulation, and notably, on the discovery of a plethora of immune checkpoints, inhibitory pathways essential for maintaining self-tolerance under physiological conditions and generating the inhibitory microenvironment for tumor to evade immune surveillance during cancer development [8, 9].

These inhibitory pathways are initiated through the ligand-receptor interactions. By far, the best characterized immune checkpoint receptors are programmed cell death protein 1 (PD-1; also known as CD279), cytotoxic T-lymphocyte-associated antigen 4 (CTLA-4; also known as CD152) and indoleamine 2,3-dioxygenase (IDO); agents targeting these molecules are either approved or being extensively tested in clinical trials for multiple solid or hematological cancers [9].

In this review, we will focus on this important strategy of immunotherapy, i.e., targeting immune checkpoints, and discuss its potential in the treatment of malignant gliomas. We will start with a brief overview on the general biology of immune checkpoints, specifically PD-1, CTLA-4, and IDO. Then we will transition to the status of different checkpoint molecules in malignant gliomas, which provides the rationale to targeting these molecules. Finally, we will review the pre-clinical and clinical trials involving the therapies targeting these immune checkpoints.

## MALIGNANT GLIOMA

Malignant gliomas are histologically heterogeneous glia-derived tumors that infiltrate the stromal tissues. In 2016, the World Health Organization (WHO) published the new classification of CNS tumors, which, for the first time, combines molecular and histological features to identify many tumor entities [10]. Following this classification system, diffuse gliomas are divided into grade II/III astrocytic tumors, grade II/III oligodendrogliomas, grade IV glioblastomas, and the related diffuse gliomas of childhood. Both grade II diffuse astrocytomas and grade III anaplastic astrocytomas are further divided into isocitrate dehydrogenase (IDH)-wildtype, IDH-mutant and NOS categories. Glioblastomas include: IDH-wildtype glioblastoma; IDH-mutant glioblastoma; and NOS glioblastoma. The NOS designation means that insufficient information is available to assign tumors to the relevant genetic parameter.

The central nervous system (CNS) was once considered immune-privileged, deficit in normal immunological functions, due to its specific anatomical and physiological features: the presence of the blood brain barrier allowing for selective entry of immune cells, the absence of lymphatic vessels or lymph nodes, the critical immune organs in the periphery, the low numbers of traditional antigen-presenting cells (APCs) including dendritic cells (DCs) and macrophages, and the lack of naive T cells in CNS [11, 12]. Nevertheless, recent progresses in neurobiology and neuroimmunology suggest that although challenging, immunotherapy holds extraordinary promises in CNS malignancies. Several recent publications convincingly demonstrated the presence of functional lymphatic vessels within the meningeal compartment [13–15], not only supporting the early descriptions that lymphatic systems exist in the brain [16–18], but also revealing novel routes that enable the communications of glioma antigens and immune cells between the brain and other immune components. Therefore, the glioma antigens may first enter the cerebrospinal fluid (CSF) through perivascular spaces termed Virchow-Robin spaces [19]. Due to the lack of secondary lymphoid tissues in the brain parenchyma, the peripheral lymphoid tissue may be the starting point for initiating tumor-specific immune responses; that is, the antigens may be transported into deep cervical lymph nodes through the newly discovered dural lymphatic, and then be presented by APCs in peripheral lymphoid tissues [13, 20, 21]. The tumor-specific lymphocytes cross the choroid plexus into CSF, are re-stimulated by local APCs, and ultimately traffic through the blood-brain barrier and/or Virchow-Robin spaces into brain parenchyma to mount an efficient immune attack on tumors. Meanwhile, specific chemokines may also play important roles in recruiting glioma-specific tumor-infiltrating lymphocytes (TILs) [21, 22].

Furthermore, the immunosuppressive state observed in patients with malignant gliomas corroborate the significance of immune system in disease development. Locally, malignant gliomas enrich their microenvironment with immunosuppressive factors such as the transforming growth factor beta (TGF-β) and vascular endothelial growth factor (VEGF), both suppressing DC maturation and inhibiting T cell proliferation and cytotoxicity [23, 24]. The reduced absolute counts of CD4+ T cells and increased proportion of immunosuppressive regulatory T cells (Tregs) further nurture the suppressive microenvironment [25]. Systematically, the old age of patient population, cytotoxic chemotherapy and other therapies including corticosteroids all contribute to the deficiency of adaptive immune responses [11]. Hence, the reverse of immunosuppression and the initiation of anti-tumor immunity hold great promise to limit tumor progression and improve patient outcomes for malignant gliomas.

## IMMUNE CHECKPOINTS AND MALIGNANT GLIOMA

The accurate execution of each step of immune regulation is fine-tuned by the balance between stimulatory and inhibitory signaling, both of which initiates with membrane receptors, also known as immune checkpoints. Given their accessibility on cell membranes and significance in regulating immune responses, the immune checkpoint receptors and their corresponding ligands become the best druggable targets for immune regulation. Therefore, in contrast to most antibodies currently used for cancer therapy, drugs targeting immune checkpoints do not target tumor cells directly, but instead act on lymphocytes to boost their endogenous anti-tumor activity [26, 27].

Of the many immune checkpoint receptors under intensive investigation, the three best characterized ones in the context of clinical cancer immunotherapy are PD-1, CTLA-4 and IDO, which function by different mechanisms, as detailed below.

### PD-1 and its ligands

**Figure 1:**
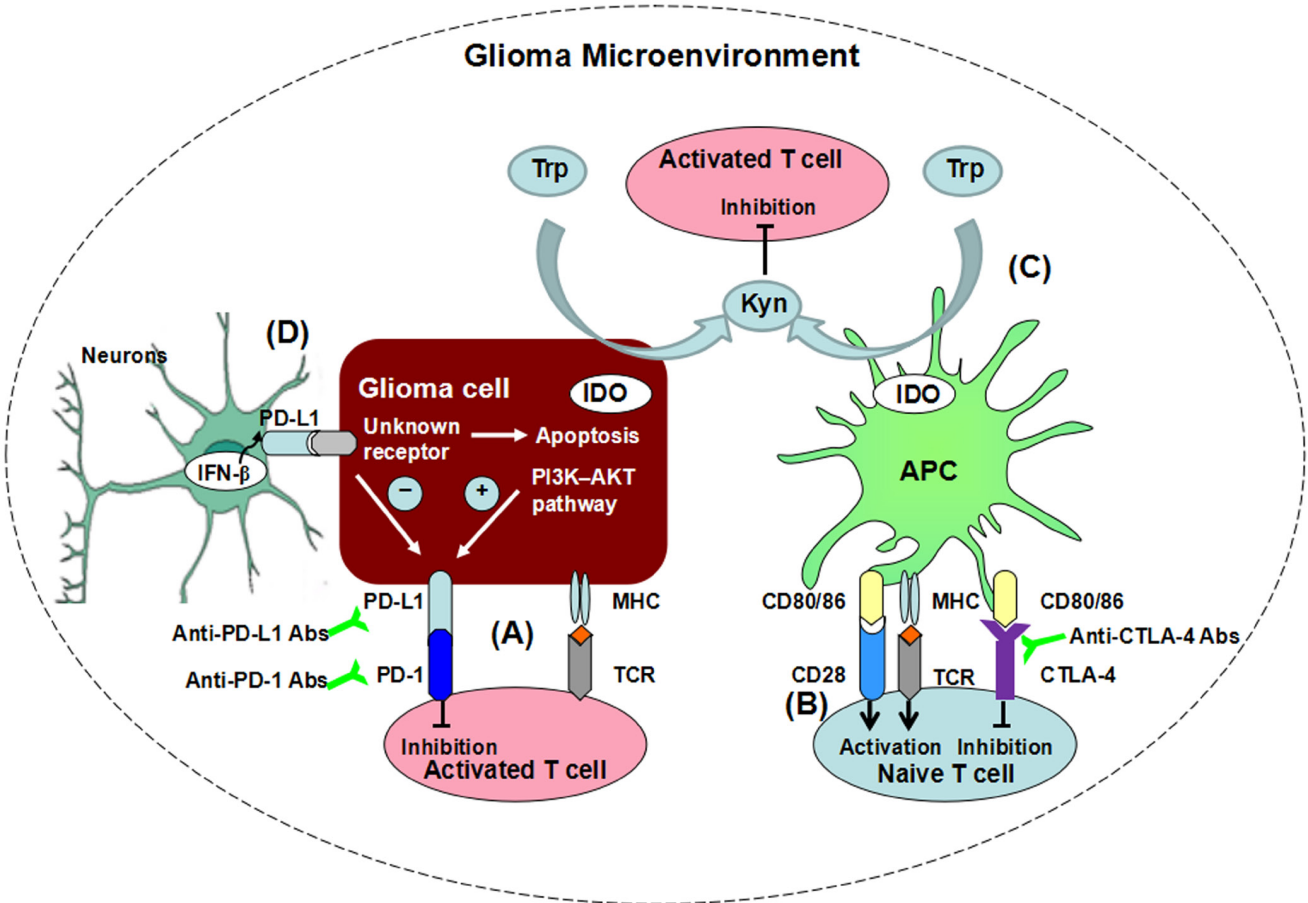
Immune checkpoints suppress T cell function in glioma microenvironment through differential mechanisms **A.** The expression of PD-L1 on glioma cells is dependent on the PI3K-AKT pathway. PD-L1, upon engagement to its receptor PD-1 on T cells, inhibits activated T cell functions within the tumor microenvironment. **B.** CTLA-4 inhibits T cell activation. The co-stimulatory molecules from APCs, CD80 and CD86 bind to both stimulatory receptor CD28 and inhibitory receptor CTLA-4, yet with lower affinity to the former than to the latter. Therefore, CTLA-4 suppresses T cell activation via competitive inhibition. **C.** The maintenance, and function of T cells require adequate Trp levels, but IDO from tumor cells catabolizes Trp to numerous metabolites, such as Kyn. The decrease of Trp suppressed T cell activation. Meanwhile, the metabolites, such as Kyn can induced T cell apoptosis. **D.** In neurons surrounding glioma tissue, the expression of PD-L1 is induced by endogenous production of IFN-β. The neurons have the capability to inhibit proliferation of glioma cells and induce its apoptosis. Meanwhile, PD-L1+ neurons reduce the PD-L1 expression on glioma cells.

**Figure 2:**
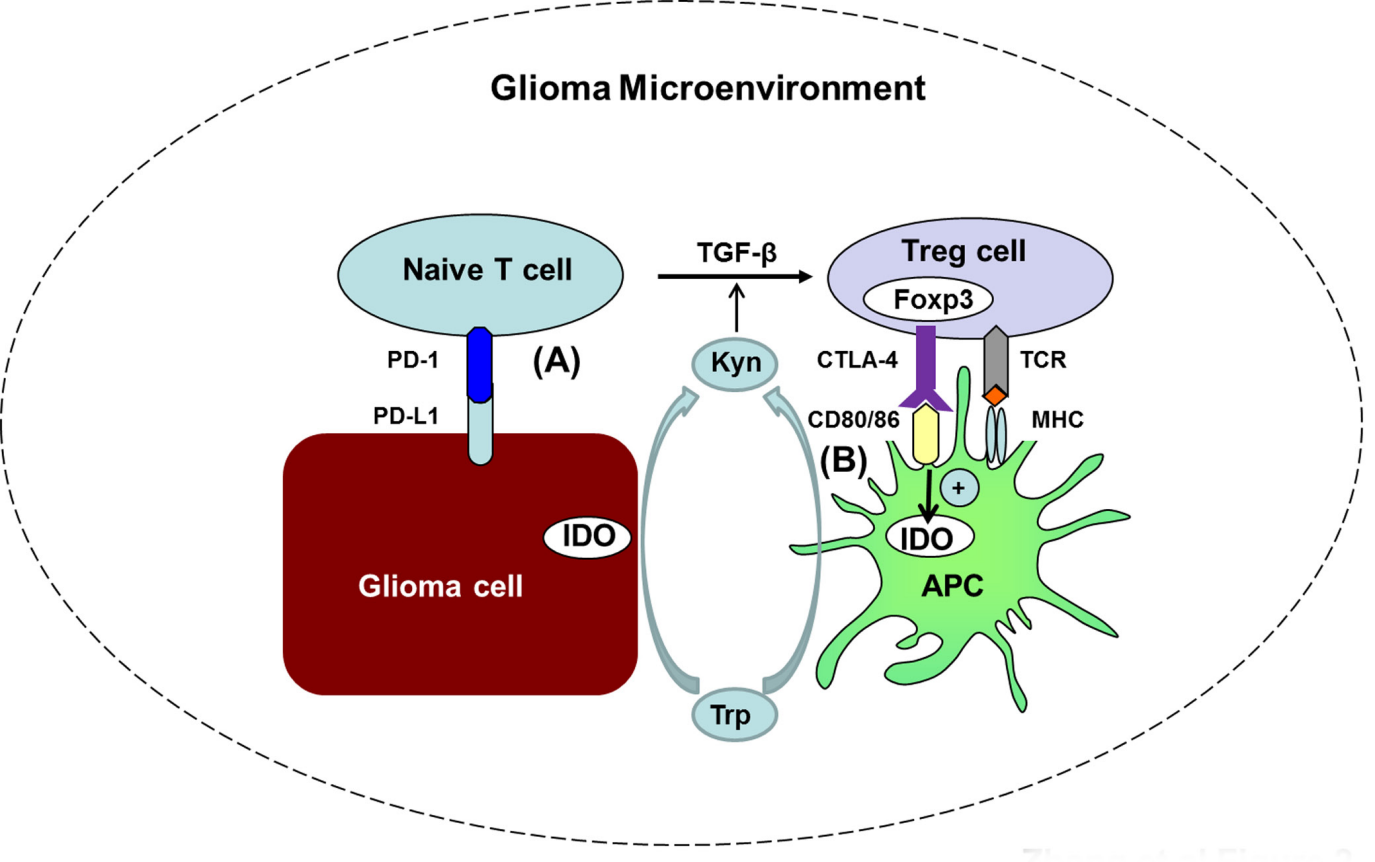
The diversified mechanisms by which various immune checkpoints promote each other, activate Tregs, and contribute to the immunosuppressive microenvironment in glioma **A.** IDO from tumor cells catabolizes Trp to Kyn. PD-1/PD-L1 pathway and Kyn could induce FoxP3 expression and promote Treg conversion with the assistance of TGF-β. **B.** FoxP3 controls CTLA-4 expression by Tregs. The CTLA-4 on Tregs binds to CD80/86, up-regulates the expression of IDO, down-regulates the expression of CD80 and CD86 on APCs, and enhances the suppressive functions of Tregs.

PD-1 is an inhibitory receptor mainly expressed on activated T cells, including CD4+ and CD8+ T cells [28, 29]. It is also detectable on other lymphocyte subsets, including natural killer (NK) cells and B cells [30, 31]. The elevated expression of PD-1 has been detected in diverse advanced human cancers, such as melanoma, prostate cancer, and renal cell carcinoma [32–34]. The immunosuppressive activities of receptor PD-1 are initiated upon binding to one of its two ligands, PD-L1 or PD-L2, and mediated through multiple mechanisms [28, 35–37]. First, upon ligand engagement, PD-1 recruits the Src homology2 domain-containing tyrosine phosphatase (SHP2), which inhibits the phosphorylation of PI3K, blocks the PI3K-Akt pathway, and suppresses T cell activity [28, 38]. This signaling is activated during antigen presentation to T cells by APCs as well as through T cell-target cell interaction. Some cancer antigens persistently up-regulate PD-1 expression in T cells, which is recognized as a mechanism termed cognate antigen-specific T cells exhaustion (Figure 1A) [39]. Second, PD-1 is highly induced on Tregs upon engagement by PD-L1, which plays a significant role in Treg development and sustaining their suppressive functions (Figure 2A) [40].

The PD-1 ligands, PD-L1 (also known as B7-H1 or CD274) and PD-L2 (also known as B7-DC and CD273) both belong to the B7 family. Through the engagement of PD-1, PD-L1 reduces IFN-γ production by activated T cells and leads to T-cell exhaustion or anergy. Furthermore, PD-L1 drives the differentiation of naive CD4+ T cells into induced Tregs, and maintains their suppressive function [40]. In addition to PD-1, PD-L1 also binds to CD80. The interaction between PD-L1 and CD80 initiates negative signals that inhibit T cell function and cytokine production [41, 42]. PD-L1 was reported to be highly expressed on malignant tumor cells, such as glioma, ovarian, melanoma and lung cancers [43–46]. Two mechanisms have been revealed to regulate PD-L1 expression by tumor cells: innate immune resistance and adaptive immune resistance [47]. The innate immune resistance refers to the finding from certain lymphomas and gliomas that the PD-L1 expression is up-regulated by constitutive oncogenic signaling in the tumor cells, including the PI3K-Akt pathway and constitutive anaplastic lymphoma kinase (ALK) signaling, both independent of inflammatory signals from the tumor microenvironment [48, 49]. Adaptive immune resistance is observed in other tumors including melanoma, where interferons, predominately IFN-γ, up-regulate the expression of PD-L1. The heterogenous expressions of PD-L1 within the tumor tissue are regions of lymphocytes infiltration as part of the immune responses [50, 51]. Other than tumor cells, PD-L1 is also expressed on stromal cells and macrophages within the tumor microenvironment [52–54].

PD-L2, a second ligand for PD-1, is exclusively expressed on APCs including DCs and macrophages under homeostasis, and can be induced in a variety of other immune cells or non-immune cells in response to microenvironmental stimuli [55]. In certain subsets of B cell lymphomas, such as Hodgkin's lymphoma, follicular cell B cell lymphoma and mediastinal B cell lymphoma, PD-L2 is highly up-regulated [56]. The up-regulation of PD-L2 in lymphomas may result from the gene fusions between the class II major histocompatibility complex (MHC) transactivator (CIITA) and PD-L2 [57]. The downstream signaling or biological functions from PD-L2 interacting with PD-1 are not clear, although studies suggest that PD-L2, through the engagement of PD-1, could inhibit T-cell responses [55, 58]. Besides, it is believed that PD-1 is not the only receptor for PD-L2, since PD-L2 mutant incapable of binding to PD-1 still impact T-cell functions [59].

### PD-1 and glioma

A recent study showed that the up-regulation of PD-1 on peripheral blood T cells of glioma patients correlates with disease progression. The proportion of PD-1+ cells among peripheral blood CD4+ and CD8+ T cells in glioma patients is higher than that in healthy controls. The CD4+ T cells from astrocytoma patients, the most malignant glioma, express the highest proportion of PD-1 compared with other types [60]. Besides the T cells in peripheral circulation, the frequency and expression level of PD-1 are also significantly higher on glioma-infiltrating CD4+ effector memory T cells, when compared with cells from healthy donors [61]. Functionally, PD-1 expression is closely related to the defects of IFN-γ production from glioma-infiltrating CD4+ effector memory T cells. Further analysis revealed that myeloid-derived suppressor cells (MDSCs), a population of myeloid precursors, are significantly accumulated in patients with glioma and contribute to tumor progression. Glioma-derived MDSCs expressed high levels of PD-L1. When co-cultured with T cells, glioma-derived MDSCs can up-regulated PD-1 expression on CD4+ T effector memory T cells. Consequently, MDSCs in glioma patients contribute to the functional exhaustion of T cells through PD-1/PD-L1 pathway [61].

For patients with GBM, the tumor-infiltrating Tregs significantly correlate with worse prognosis. PD-1 is highly induced on Tregs upon engagement by PD-L1 expressed on glioma, which plays a significant role in Treg development and sustaining their suppressive functions and is responsible for immune escape of glioma [40, 62]. Thus, blockade of the PD-1 signaling may inhibit the production of Treg, and specifically boost anti-tumor immune responses [62].

### PD-L1 and glioma

PD-L1 is highly expressed in human malignant gliomas, and its levels significantly correlated with the glioma grade. Recent studies showed that tumor cells from Grade IV gliomas, anaplastic astrocytoma and GBM, present intense positive staining for PD-L1, which was reduced in oligodendroglioma and diffuse astrocytoma (Grade II and III gliomas), and no positive staining of PD-L1 was detected in the tumor-free CNS tissue [46, 63, 64].

The reported positivity of PD-L1 expression in GBM ranges from 61% to 88% [65], potentially due to the differences in sample size, tissue sampling strategies, antibodies and staining protocols, methods for evaluating staining patterns, and assignment of cut-off values. Tissue sampling may critically affect the analysis on PD-L1 expression. Insufficient sampling may not include the PD-L1-positive areas of heterogeneous tumors, generating false negative results and the lower percentage of PD-L1-positive glioblastomas. Therefore, there is an urgent need to standardize staining procedures and evaluation methods to analyze PD-L1 expression in tissue samples [66].

In malignant glioma, PD-L1 expression in tumor cells is controlled through the innate immune resistance mechanism, by the constitutive oncogenic signaling, PI3K-AKT pathway in tumor cells [49]. It is demonstrated that PD-L1 expression is elevated posttranscriptionally in glioma cells after loss of phosphatase and tensin homolog (PTEN) and activation of PI3K pathway (Figure 1A).

Other than glioma cells and tumor-infiltrating lymphocytes [67], PD-L1 expression is also detected on neurons surrounding glioma tissue [68, 69]. There are significant correlations between the clinical outcomes and the level of PD-L1 on neurons from GBM patients. The Up-regulation of PD-L1 on neurons in tumor adjacent brain tissue was associated with prolonged survival, whereas the loss of neuronal PD-L1 was involved in high PD-L1 expression on glioma tissue and poor prognosis. In light of these findings, the expression of PD-L1 is induced by endogenous production of IFN-β in neurons surrounding glioma tissue (Figure 1D). Therefore, the expression of PD-L1 in tumor adjacent brain tissue is important for the survival of patients with malignant glioma [70]. Mutual PD-L1 regulation between tumor-adjacent brain tissue and tumor tissue is a potential prognostic biomarker for GBM.

### CTLA-4 and glioma

CTLA-4, also known as CD152, is a member of the immunoglobulin superfamily and the first co-inhibitory molecule identified [71]. CTLA-4 is not expressed by naive T cells, but rapidly induced in both CD4+ and CD8+ T cells upon T-cell activation.

The predominant functions of CTLA-4 are attributed to the effects on the two major subsets of CD4+ T cells: suppression of helper T cell activity and enhancement of Treg immunosuppression [72]. The ligands of CTLA-4 are CD80 and CD86, both of which also signal through the co-stimulatory receptor CD28 and induce T-cell activation. Since CTLA-4 has a much higher affinity for both ligands than CD28, it effectively inhibits the ligand engagement to CD28, transduces inhibitory signals, and suppresses T-cell activation (Figure 1B) [73–75]. Forkhead box P3 (FoxP3), a forkhead transcription factor, controls CTLA-4 expression (Figure 2B). In return, when CTLA-4 is induced in Tregs, CTLA-4 mediates the down-regulating of co-stimulatory molecules, CD80 and CD86 on DCs, and thus reduces the potency of APCs to activate other T cells, critically controlling the immunosuppressive activity of Tregs (Figure 2B) [76]. In addition, CTLA-4 expressed by Tregs could bind to CD80/86 on DCs with high affinity, and then deliver a signal that induces IDO expression, and triggers the IDO activity in DCs (Figure 2B) [77].

A study in glioma patients showed that CTLA-4 is highly expressed on T cells, specifically the effector CD4+T cells and Tregs [78]. More importantly, the expression of CTLA-4 is correlated with the glioma patients’ progression. In patients with malignant glioma, the CD4+T-cell count decreased dramatically in the peripheral blood, spleen, and cervical lymph nodes, while Tregs significantly accumulated in the tumor microenvironment; both collectively contributing to the immunosuppressive milieu of the tumor [25, 79]. Consistent observations were also made in mouse models of gliomas [80]. Consequently, when CTLA-4 inhibitor was applied, CD4+T-cell proliferative capacity was enhanced, and the Treg/CD4+ T ratio was lowered although Treg numbers were not affected, which restored T-cell functional defects and produced stronger anti-tumor response in vitro. Based on these findings, we speculate that the biological activities of CTLA-4 in malignant glioma are carried out preferentially through the inhibition of CD4+ T cell activity.

In response to DC vaccines, an innovative therapeutic method for patients with glioma, the down-regulation of CTLA-4 on peripheral blood lymphocyte closely correlates with good prognosis in glioma patients, further supporting the significance of CTLA-4 in regulating anti-tumor immune responses [78].

### IDO and glioma

IDO is expressed by various tumor cells and DCs. Although not an immune checkpoint in the classical sense, IDO mediates several inhibitory pathways in various tumor types and suppresses anti-tumor immunity as a mammalian cytosolic enzyme [81].

IDO is a tryptophan (Trp) catabolic enzyme responsible for Trp degradation through the kynurenine (Kyn) degradation pathway [82–84]. IDO from tumor cells catabolizes Trp to numerous metabolites, including Kyn, 3-hydroxykynurenine (3-HK), and 3-hydroxyanthranilic acid (3-HAA). The maintenance and function of T cells require Trp [85]. Therefore, the Trp degradation mediated by IDO suppresses T cell activation. Meanwhile, the metabolites, such as Kyn, 3-HK, and 3-HAA induce T cell apoptosis (Figure 1C) [86]. In addition, when combined with TGF-β, Kyn could induce FoxP3 expression, and stimulate Treg formation [87–89]. And IDO expressed by tumor cells also induces Treg infiltration into tumor microenvironment (Figure 2B). Together, these mechanisms exacerbate immune suppression associated with cancer development.

In glioma, IDO expressed in glioma cells plays an important role in inhibiting T cell functions and contributes to Treg accumulation [90, 91]. Under normal physiology, the CNS parenchyma does not express IDO [92]. 90% of GBM, however, shows positive expression of IDO [90, 93], which is further boosted in low grade glioma, suggesting that IDO expression level correlates with the severity of glioma [94]. Moreover, GBM patients with higher expression of IDO have a worse prognosis [90]. However, a recent study showed that only 8% of GBM expressed IDO [95]. Considering that the antibodies used in these studies, although different from one another, were all well controlled for the detection specificity, these studies suggest that alternative splice variants and/or posttranslational modifications of IDO protein in GBM samples may result in antigenic variations, and thus the dichotomous findings of IDO in GBM.

### Other immune checkpoints and glioma

In addition to PD-1, CTLA-4, and IDO, other immune checkpoints are also involved in the occurrence and development of glioma, including T cell membrane protein-3 (TIM-3; also known as HAVcr2), killer inhibitory receptors (KIR), and V-domain Ig-containing suppressor of T cell activation (VISTA). Similar to PD-1, CTLA-4, and IDO, these immune checkpoints inhibit lymphocyte activity and/or induce lymphocyte anergy, and thus are ideal targets for glioma immunotherapy. Blocking antibodies for these immune checkpoints have shown specific anti-tumor activities in animal models, and some are being tested in clinical trials.

TIM-3 is mainly expressed on activated T cells, NK cells, and monocytes [96–98]. It binds to galectin-9, through which it induces Th1 cell death and suppresses the anti-tumor immune response [96]. The significance of the TIM-3 has been demonstrated in different cancer models, including colon cancer, mammary carcinoma, and melanoma [99, 100]. In glioma patients, the expression of TIM-3 on CD4+T cells and CD8+T cells is significantly elevated than in healthy controls, and the higher expression level of TIM-3 on T cells is associated with tumors of higher grades [101].

KIRs (Killer inhibitory receptors) are a collection of inhibitory receptors that can down-regulate NK cells cytotoxic activity and inhibit cytokine secretion of NK cells [102]. Many KIRs are also expressed on T cells and APCs. Activation of KIRs in T cells suppresses the cytolytic activities of these cells. Consistently, expressions of KIRs are detected in multiple cancer types and correlate with poor prognosis [103, 104]. Among KIRs, CD94/NKG2A is an NK inhibitory receptor expressed by most the astrocytoma-infiltrating T cells [105]. An anti-NKG2A antibody (IPH2201) is undergoing phase I/II trial testing in HNSCC patients (NCT02331875) [104]. This antibody could be applied into glioma clinical trial. Many other anti-KIR mAbs are also being tested in anti-tumor clinical trials. A phase I trial of anti-KIR (IPH2101) in patients with acute myelogenous leukemia has been completed. Several studies involving anti-KIR (lirilumab) combined with anti-PD-1 (clinical trial NCT01714739) or with anti-CTLA4 (clinical trial NCT01750580) are undergoing on patients with hematologic and solid cancers. These trials will investigate the anti-cancer effect by simultaneously boosting both the innate immune activity, via anti-KIR, and the adaptive immune activity via anti-PD-1 or anti-CTLA4.

The VISTA is a member in the CD28 receptor family and is mainly expressed on myeloid and granulocytic cells, including naive T cells, NK cells, macrophages, and DCs, but not on B cells [106]. Through the interaction with an unknown receptor on T cells, VISTA negatively inhibits T cell responses [107]: not only T-cell proliferation, but also the expression of activation markers and the production of cytokines. In vitro, VISTA induces the development of Tregs with the help of TGF-β [108]. In line with its biological activities, VISTA-KO mice are resistant to the development of GL261 glioma [109]. In addition, when combined with a cancer vaccine, VISTA blockade inhibits tumor growth [107]. In a glioma mouse model, GL261 cells were directly injected into the left cerebral hemisphere of WT or VISTA-KO mice and the tumor growth was monitored and recorded using imaging technique system all the time. WT mice tumors died within 35 days after GL261 inoculation, while no tumor developed in approximately 20% of VISTA-KO mice. Therefore, targeting VISTA may present potent therapeutic efficacy on glioma.

Other immune checkpoint molecules, including lymphocyte activation gene-3 (LAG-3; also known as CD223), 2B4 (also known as CD244), B and T lymphocyte attenuator (BTLA; also known as CD272), have not been fully explored for their biological activities and functions in glioma. Given their importance as “next-generation” cancer immunotherapy, further studies should be directed on their significance and therapeutic potentials in glioma.

## IMMUNE CHECKPOINTS BLOCKADES IN CLINICAL TRIALS FOR MALIGNANT GLIOMA

The preclinical findings suggest that immune checkpoints are optimal targets for cancer immunotherapy, which stimulated the development of inhibitors targeting these checkpoint receptors and/or their ligands, including antibodies for PD-1 (pembrolizumab, nivolumab, and pidilizumab) [110–112], PD-L1 (MEDI4736, MPDL3280a, and MDX-1105) [113, 114], CTLA-4 (ipilimumab and tremelimumab) [115–117], and IDO (indoximod and INCB024360)[118]. Although some of the antibodies have been approved by US Food and Drug Administration (FDA) to treat melanoma and non-small lung cancer, they are still under intensive investigations for the treatment of malignant gliomas (Table 1).

**Table 1:** Clinical trials on checkpoint-blocking antibodies in patients with glioma

Target	Intervention	Clinical Trials No.	Phase	Condition
PD-1	Pidilizumab	NCT01952769	Phase I/II	Diffuse pontine glioma
	Pembrolizumab + MRI-guided laser ablation	NCT02311582	Phase I/II	Recurrent malignant glioma
	Nivolumab + DC Vaccines	NCT02529072	Phase I	Recurrent brain tumors
	Pembrolizuma + Adenovirus	NCT02798406	Phase II	Recurrent glioblastoma or gliosarcoma
	Nivolumab + FPA008	NCT02526017	Phase I	Advanced solid tumors, including glioma
	Nivolumab +Galunisertib	NCT02423343	Phase I/II	Advanced solid tumors, including glioma
	Nivolumab + anti-LAG-3 or anti-CD137	NCT02658981	Phase I	Recurrent glioblastoma
CTLA-4 & PD-1	Ipilimumab/nivolumab, or both + Temozolomide	NCT02311920	Phase I	Newly diagnosed glioblastoma or gliosarcoma
	Nivolumab ± Ipilimumab vs Bevacizumab	NCT02017717	Phase III	Recurrent glioblastoma
PD-L1	MEDI4736 ± radiotherapy vs MEDI4736 + Bevacizuma	NCT02336165	Phase II	Glioblastoma
IDO	Indoximod + Temozolomide + Bevacizumab + Radiation	NCT02052648	Phase I/II	Adult patients with primary malignant brain tumors
	Indoximod + Temozolomide + Conformal Radiation	NCT02502708	Phase I	Pediatric patients with primary malignant brain tumors

### PD-1 blockade

Preclinical studies demonstrated the potential of PD-1 antibodies enhanced the anti-tumor immune responses in a variety of glioma mouse models [60]. At present, at least three PD-1 blocking antibodies are in clinical trials for glioma patients, including pembrolizumab (a humanized IgG4 antibody), nivolumab (a fully human IgG4 antibodies), and pidilizumab (a humanized IgG1 antibody). Pembrolizumab and nivolumab have been approved by the US FDA for melanoma, non-small cell lung cancer, renal cell cancer, Hodgkin's lymphoma, squamous-cell carcinoma of the head and neck, and bladder cancer [119–124].

For glioma, a Phase I/II clinical trial of pidilizumab (NCT01952769) started in February 2014 in patients with diffuse intrinsic pontine glioma, and is expected to end in November 2018. In this study, treatment-related toxicity and progression-free survival will be recorded to evaluate the safety, toxicities, and efficacy of the antibody. Meanwhile, a phase I/II study of pembrolizumab is ongoing among patients with recurrent malignant gliomas to evaluate the maximal tolerated dose and progression-free survival of pembrolizumab in combination with radiotherapy. This study received the first patient in December 4, 2014, and is expected to be completed in June 2018. (Table 1)

Recently, several studies reported the safety/tolerability of the anti-PD-1 antibody nivolumab, alone or in combination with other treatments in patients with high-grade glioblastoma (GBM) [125, 126]. A randomized trial reported at the ASCO 2015 showed that nivolumab alone or combined with ipilimumab did not generate any serious and specific toxicities in 20 patients with recurrent GBM. Another study reported on the 20th Annual Scientific Meeting of the Society for Neuro tested the concurrent therapy of pembrolizumab or nivolumab with different treatment regimens on 12 patients with recurrent high grade GBM and found no toxicities [127]. Thus, PD-1 inhibitors can be administered safely to patients with other therapies including re-irradiation. An ongoing phase III study (NCT02617589 ) was designed to compare overall survival of nivolumab versus TMZ, each in combination with radiation in newly diagnosed GBM patients with unmethylated MGMT. Therapeutic effects will be judged by tumor progression, safety and tolerability, and survival [128]. There were no serious and specific toxicities associated with PD-1 inhibitors among all clinical trials. Therefore, PD-1 inhibitors can be safely administered to patients with high-grade gliomas in combination with other treatments such as radiation.

### PD-L1 blockade

PD-L1 is another promising target for disrupting the PD-1/PD-L1 pathway. Three PD-L1 clinical agents have demonstrated clinical activity in several solid tumor types, including MEDI4736, MPDL3280a, and MDX-1105 antibodies [129, 130]. MEDI4736 and MPDL3280A are two kinds of Fc-modified human IgG1 antibodies. MDX-1105 is a fully human IgG4 antibody. Among these agents, MEDI4736 is being tested in patients with GBM (NCT02336165) (Table 1), where GBM patients are divided into three group: patients with newly diagnosed GBM are given with MEDI4736 every 2 weeks combined with radiotherapy; Bevacizumab-naïve patients with recurrent GBM receive MEDI4736 every 2 weeks as monotherapy or in combination with bevacizumab every 2 weeks; bevacizumab-refractory subjects with recurrent GBM are administered MEDI4736 every 2 weeks in combination with continued bevacizumab. Clinical efficacy and quality of life are being recorded in this phase II study.

### CTLA-4 blockade

The anti-CTLA-4 monoclonal antibodies are the first immune checkpoint inhibitors in clinical trial. In 2000, the blockades of CTLA-4, ipilimumab (a fully human IgG1 antibody), and tremelimumab (a fully human IgG2 antibody) entered clinical trials. Ipilimumab is the first agent approved by the US FDA for the treatment of melanoma in 2010 [131].

A phase I and phase III clinical trials of ipilumumab, in combination with another immunomodulatory nivolumab in GBM are currently ongoing (NCT02311920, NCT02017717) (Table 1). The phase I clinical trial mainly evaluates the dose limiting toxicities of ipilimumab, nivolumab, and their combination in patients with newly diagnosed GBM who receive temozolomide. The phase III clinical trial mainly evaluates the efficacy and safety of nivolumab, with or without ipilimumab, and compares the efficacy and safety of nivolumab versus bevacizumab in GBM patients at different stages of treatment.

Neuro-Oncology recently published a clinical trial on anti-CTLA-4 monoclonal antibody. In this study, seven patients (age range: 39 to 66 years) with high-grade gliomas (one with recurrent grade 3 and 6 with grade 4 glioma) were treated with ipilimumab between June 2012 and April 2014 [132]. Five patients received other therapies, including bevacizumab, in combination with ipilimumab. Of the 7 patients, 3 died within 5 months of the treatment and 4 have survived for 3.2-23.3 months. Specifically, one patient with anaplastic astrocytoma achieved recurrence-free after 3.2 months of treatment, and has stable disease with improvement in functional status, steroid requirement and other clinical symptoms. The up-regulation of granzyme B expression was also found through immunohistochemistry, indicating a stronger immune response. Accordingly, a subset of patients with recurrent high-grade glioma may benefit from anti-CTLA-4 treatment.

### IDO blockade

Several inhibitors targeting IDO, including competitive inhibitor (such as 1-MT), and uncompetitive inhibitor (such as exiguamine A) have been developed. 1-MT has two optical isomers, left-handed form (L-1-MT) and right-handed form (D-1-MT). The phase I clinical trial of D-1-MT (indoximod) combined with chemotherapy has completed [83, 133]. The safety and anti-tumor activity have been demonstrated. Meanwhile, INCB024360, a new-generation IDO inhibitor, has entered clinical tests.

The clinical trials of indoximod on glioma are undergoing (NCT02052648 and NCT02502708), which will lay the foundation for combination therapy of indoximod together with radiation and temozolomide for glioma patients, including adult patients and children. The phase I/II clinical study (NCT02052648) of indoximod are conducted among adult patients with recurrent malignant brain tumors. In this study, the patients will receive the combination of indoximod and temozolomide with either stereotactic radiation or bevacizumab. The safety and the optimal dose of indoximod will be identified. This clinical trial brings IDO-based immunotherapy into glioma therapy. In another phase I clinical trial (NCT02502708) using indoximod in combination with temozolomide plus radiation to treat children with brain tumors, the test dose of indoximod will be from 12.8 mg/kg/dose to 22.4 mg/kg/dose; the safety and tolerability will be also assessed by incidence and severity of adverse events. (Table 1)

## THE PRECLINICAL COMBINATION STUDY FOR MALIGNANT GLIOMA

Cancer employs multiple mechanisms to foster its own growth and metastasis [134]. Therefore, the combination therapy targeting distinct mechanisms simultaneously offers advantages over single-drug therapy. In this section, we will discuss the latest combination studies on immune checkpoint inhibitors in mouse model of malignant gliomas.

### Combination of different immune checkpoint blockades

The early preclinical studies have shown that the combination of PD-1/PD-L1 and CTLA-4 blockades is more than two times as effective as either alone to eradicate tumor cells. The synergistic anti-tumor activity has been verified in mouse models of melanoma and colon adenocarcinoma [135, 136].

Basic immunologic observations support the notion that PD-1/PD-L1 pathway, CTLA-4, and IDO regulate T-cell responses and promote tumor immunosuppression through overlapping as well as non-redundant mechanisms [40, 75, 76]. In addition, these immune checkpoint molecules regulate each other and further promote the immunosuppression [137]. PD-1/PD-L1 pathway and IDO could induce FoxP3 expression and promote Treg differentiation. FoxP3 controls CTLA-4 expression in Tregs [40, 84]. The CTLA-4 could bind to the CD80/86, and up-regulate the expression of IDO [77]. Consistently with their functions, PD-1/PD-L1, CTLA-4, and IDO are highly expressed in glioma microenvironment. When mAbs for CTLA-4 and PD-L1, together with the IDO inhibitor, 1-MT, were administered to mice bearing glioma, Treg levels in glioma microenvironment decreased significantly, and the combination was more effective than the single agent [138].

To achieve the maximal immune suppression during cancer development, immune checkpoint molecules are frequently co-expressed on lymphocytes. For example, LAG-3 and TIM-3 are commonly co-expressed with PD-1 on tumor-infiltrating T cells and work synergistically to regulate immune functions [139, 140], suppressing the proliferation of T cells and the production of cytokines such as IL-2, TNF, and IFN-γ. Consequently, the combined use of inhibitors for LAG-3/TIM-3 and PD-1 was more effective in stimulating anti-tumor immunity than blocking each individual component in different tumor mode [139, 140]. Similarly, the combination of anti-TIM-3 and anti-PD-1 with stereotactic radiosurgery has shown the potential to enhance anti-tumor immunity and prolong survival in a mouse model of glioma [141].

### Immune checkpoint blockades combined with traditional therapy

The traditional therapies for glioma mainly involve surgical removal, chemotherapy, and radiotherapy, which, however, could not eliminate the tumor completely due to the extensive tumor infiltration into the surrounding stroma. In this regard, immunotherapy provides a promising complementary therapy in eradicating glioma.

A recent study in a mouse orthotopic GBM model showed that combining steteotactic radiation with anti-PD-1 blockade increased the tumor-infiltrating cytotoxic T cells while reduced tumor-associated Tregs, resulting in prolonged mouse survival [142]. In this study, the stereotactic radiosurgery delivers high-dose radiation in a single session, which may disrupt the immunosuppressive microenvironment without several immunologic and neurologic side effects associated with conventional radiotherapy. The anti-PD-1 blockade enhances the anti-tumor immunity, maintains the immunologic memory, and synergizes with stereotactic radiosurgery.

In the subcutaneous or intracranial glioma mouse model, oral administration of IDO inhibitors and temozolomide significantly inhibited tumor growth, prolonged survival, and presented synergistic anti-tumor effects. Additional radiation therapy in combination with IDO inhibitors and temozolomide contributes to widespread complement deposition and prolongs mouse survival [143, 144].

### Immune checkpoints blockades combined with other immunotherapy

Cancer immunotherapy continues to translate finding from immune molecular mechanisms to novel therapeutic approaches. The current immunotherapeutic approaches mainly include tumor antigen-targeted monoclonal antibodies, immune checkpoint inhibitors, cytokine therapy, and cancer vaccines. Each approach acts through a distinct mechanism, yet all work to boost anti-tumor immunity [7]. Therefore, it is expected that combining different immunotherapeutic approaches would benefit cancer treatment. Agarwalla et al. showed that sequential immunotherapy with vaccination by irradiation glioma cells producing high levels of granulocyte-macrophage colony stimulating factor followed by CTLA-4 blockade significantly improved the survival of mice with intracranial tumors [145]. Berg et al. demonstrated the potency of combining intratumoral IL-12 administration with CTLA-4 blockade in eradicating glioma cells, which was associated with the recruitment of effector T cells and the reduction of Tregs in tumor [146]. In another study, depletion of Tregs by blocking CD25 strongly enhanced the anti-tumor efficacy of DC vaccination in murine glioma model [147]. Moreover, co-administration of anti-CTLA-4 antibody and Treg depletion was used to reverse the tumor immune tolerance, further strengthened CD4+ and CD8+ effector T cells, and resulted in complete glioma eradication without autoimmunity [148]. A triple therapy including the agonist antibodies for 4-1BB (a co-stimulatory molecule expressed on T cells that can enhance the T cell-mediated rejection of tumors), CTLA-4 blocking antibodies, and focal radiation therapy, when compared to component treatment, dramatically improved the long-term tumor-free survival of mice with intracranial gliomas [149].

## FUTURE RESEARCH DIRECTION

Although blocking antibodies for immune checkpoints significantly improved the anti-tumor activities of other therapeutic approaches and prove to be a valuable treatment strategy, the high cost associated with generating a decent amount of blocking antibodies for treatment challenges their accessibility to general public. Furthermore, systemic delivery of immune checkpoint blockades may also lead to unwanted side effects, such as dose-dependent autoimmune responses. Therefore, future research should be directed, on one hand, toward improving the delivery vehicles and delivery approaches to boost the concentrations of the blocking antibodies within tumor microenvironment, reducing the therapeutic dosage and minimizing side effects, and on the other hand, toward designing novel approaches to target immune checkpoints.

The blood-brain barrier is the main obstacle for the entrance of therapeutic drugs into the brain. Recently, an advanced self-degradable microneedle patch was invented for the sustained release of anti-PD-1 antibody with minimal invasiveness and least dose of antibody required [150]. The microneedle is made up of hyaluronic acid and pH-sensitive nanoparticles encapsulating anti-PD-1 and glucose oxidase. Glucose oxidase could convert glucose to gluconic acid. The generation of gluconic acid changes the pH, induces the self-dissociation of nanoparticles and promotes substantial release of anti-PD-1 subsequently. This administration strategy has demonstrated its potential for inducing stronger immune responses compared to intratumoral injection of anti-PD-1 with the same dose in a melanoma mouse model [151]. This novel approach allows the compatibility with blocking antibodies for other immune checkpoints (such as anti-CTLA-4) as well as targeting gliomas, since the patch could be directly delivered to the capillary bed within the brain, bypassing the blood-brain barrier. Similar to the microneedle patch, osmotic mini pumps are under intensive investigations in preclinical trial for the treatment of glioma [146].

Unlike blocking antibodies, microRNAs (miRNAs) is a promising strategy to down-regulating gene expression by either inducing mRNA degradation or inhibiting mRNA translation [152]. MiR-138, a miRNA abundant in the brain, is a tumor suppressor, with its inhibition detected in multiple cancers. Both PD-1 and CTLA-4 are downstream targets for miR-138. Upon transfection into CD4+T cells, miR-138 could inhibit the expressions of CTLA-4, PD-1, and FoxP3 in T cells. In vivo, miR-138 treatment potently suppresses glioma growth, decreasing the ratio of intratumoral Treg, reducing the expression of CTLA-4 and PD-1, and leading to a 43% increase in the median survival time [153].

## CONCLUSIONS

The area on immune checkpoint blockades has witnessed rapid progression, especially the monoclonal antibodies for PD-1/PD-L1 and CTLA-4, which have been proved by FDA for melanoma and/or lung cancer as sole reagents. Meanwhile, more new reagents are under development, including those targeting LAG-3, TIM-3, and other checkpoint inhibitors. By far, no immune checkpoint blockade has been approved by the FDA for the treatment of malignant glioma. This requires the further understanding on neuroimmunology related to malignant glioma, the translation of mechanistic findings, and the design of rational combinatorial regimens involving immune checkpoint blockades.

## Abbreviations

3-HK: 3-hydroxykynurenine
3-HAA: 3-hydroxyanthranilic acid
AHR: aryl hydrocarbon receptor
ALK: anaplastic lymphoma kinase
APCs: antigen-presenting cells
BTLA: B and T lymphocyte attenuator
CNS: central nervous system
CTLA-4: the cytotoxic T-lymphocyte-associated antigen-4
DCs: dendritic cells
FoxP3: Forkhead box P3
GBM: glioblastoma multiforme
IDO: indoleamine 2,3-dioxygenase
KIR: killer inhibitory receptors
Kyn: kynurenine
LAG-3: Lymphocyte activation gene-3
MDSCs: myeloid-derived suppressor cells
MHC: major histocompatibility complex
NK cells: natural killer cells
PD-1: programmed cell death protein-1
PTEN: phosphatase and tensin homolog
SHP2: Src homology2 domain-containing tyrosine phosphatase
TGF-β: transforming growth factor beta
TILs: tumor infiltrating lymphocytes
TIM-3: T cell membrane protein-3
Tregs: regulatory T cells
Trp: tryptophan
VEGF: vascular endothelial growth factor
VISTA: V-domain Ig-containing suppressor of T cell activation
